# ATM-TCR: TCR-Epitope Binding Affinity Prediction Using a Multi-Head Self-Attention Model

**DOI:** 10.3389/fimmu.2022.893247

**Published:** 2022-07-06

**Authors:** Michael Cai, Seojin Bang, Pengfei Zhang, Heewook Lee

**Affiliations:** ^1^School of Computing and Augmented Intelligence, Arizona State University, Tempe, AZ, United States; ^2^Biodesign Institute, Arizona State University, Tempe, AZ, United States

**Keywords:** TCR, epitope, antigen, binding affinity, adaptive immunotherapy, machine learning, self-attention model

## Abstract

TCR-epitope pair binding is the key component for T cell regulation. The ability to predict whether a given pair binds is fundamental to understanding the underlying biology of the binding mechanism as well as developing T-cell mediated immunotherapy approaches. The advent of large-scale public databases containing TCR-epitope binding pairs enabled the recent development of computational prediction methods for TCR-epitope binding. However, the number of epitopes reported along with binding TCRs is far too small, resulting in poor out-of-sample performance for unseen epitopes. In order to address this issue, we present our model ATM-TCR which uses a multi-head self-attention mechanism to capture biological contextual information and improve generalization performance. Additionally, we present a novel application of the attention map from our model to improve out-of-sample performance by demonstrating on recent SARS-CoV-2 data.

## 1 Introduction

**Figure 1:**
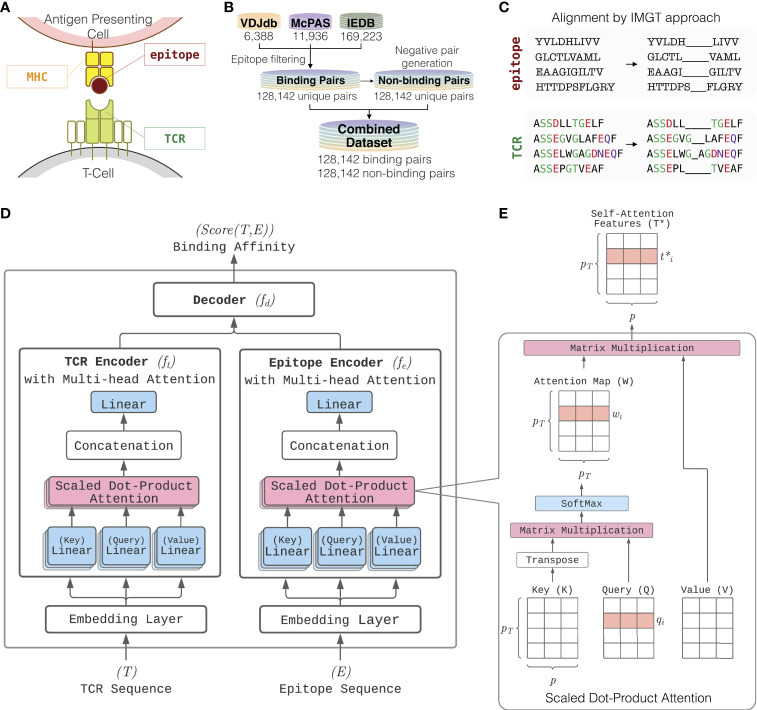
Overview of ATM-TCR. **(A)** T-cell epitope recognition. The T-cell receptor (TCR) is a protein complex on the surface of a T-cell. It recognizes epitopes displayed by MHC complex on cell surface. **(B)** The training and testing datasets consist of TCR-epitope pairs known to bind collected from VDJdb, McPAS-TCR, and IEDB. The three databases are combined into a single dataset containing unique binding pairs. The same number of negative pairs is generated through random recombination. **(C)** Each of the TCR and epitope sequences are aligned *via* IMGT approach where the paddings are inserted into the middle of each sequence until its length reaches a pre-defined value. **(D)** Model architecture of ATM-TCR. It takes a pair of TCR and epitope sequences as an input and predicts the binding affinity between the two. It consists of two parts where a TCR and epitope are processed separately. TCR and epitope are 20 and 22 amino acids long, respectively, including paddings in the middle. Each sequence is passed through a separate embedding layer which maps the sequence of one-hot amino acid vectors to a sequence of continuous feature vectors with the size 25. These features are then passed into a corresponding encoder. Each encoder makes use of the multi-head attention mechanism which allows five self-attention layers are learned in parallel. Outputs of the five self-attention layers are then concatenated and linearly transformed to obtain an encoded sequence representation. The encoded TCR and epitope sequences are then concatenated together and passed into a decoder with three linear layers of size 2048, 1024, and 2 which determines the final binding affinity score between the two. **(E)** The multi-head attention layer makes use of multiple self-attention layers called scaled dot-product attention. It utilizes the input sequence for each of its three inputs: *key* (K), *query* (Q) and *value* (V). It uses a scaled dot-product between keys and queries to obtain an attention map relating different positions of the sequence. The attention map is then used to weight values and compute a new sequence representation, denoted as self-attention features T^*^.

The hallmark of the adaptive immune system is the T cells’ ability to distinguish foreign invaders from host cells. T cells carry out this important task by utilizing their surface protein complex, called the T cell receptor (TCR) to bind to foreign peptides presented by major histocompatibility complex (MHC) molecules (also known as HLA molecules if the host is human) on host cells’ surface (illustrated in **Figure 1A**). The epitope is a specific region on a peptide to which a TCR binds to. The recognition of epitopes is a critical mechanism in immune response regulation. Therefore, unraveling the underlying principles of the TCR-epitope binding process is fundamental to developing novel clinical applications in immunotherapy (1).

One important application area is in cancer immunotherapy. Since cancer is a disease caused by many random genetic mutations, tumor cells produce “neoantigens” that are different from those produced by a patient’s healthy cells (2). Determining which TCRs bind to patient-specific neoantigens is an important question for therapy design. Also, with the current pandemic of SARS-CoV-2, the value of rapid screening for suitable candidate TCRs binding to peptides originating from infectious diseases has become clear.

Assessing which TCRs will bind to target epitopes is challenging, as there are over 10^15^ rearrangements of the VDJ genes in T-cells, each possible recombination resulting in a distinct TCR (3). Furthermore, the relationship between TCRs and epitopes is many-to-many, meaning it is possible for TCRs to bind to multiple epitopes while epitopes can also bind to multiple TCRs (4) With such an extremely large search space, manually testing candidate TCRs against a particular epitope becomes an infeasible solution. The ability to computationally infer the binding affinity of TCR-epitope pairs is crucial in rapid development of truly personalized immunotherapy.

The advent of large-scale public databases containing epitope-specific TCR sequences such as VDJdb (5), McPAS-TCR (6), and the Immune Epitope Database (IEDB) (7) has opened a door to computational methods for determining the immunogenicity of given epitope sequences. Machine learning based solutions such as NetTCR (8), TCRex (9), TCRGP (10), and ERGO (11) have been proposed to utilize these large datasets. The aforementioned methods aim to predict binding affinity of TCR-epitope pairs as a binary classification problem. Currently all available computational methods focus only on linear epitopes and do not consider conformational epitopes (epitopes consisting of multiple change of residues that are discontinuous). NetTCR utilizes convolutional neural networks followed by multiple dense layers to learn interaction between TCRs and epitopes presented by the most common human allele HLA-A*02:01. TCRex builds an epitope-specific prediction model using the random forest classification algorithm composed of a series of decision trees. TCRGP focuses on utilizing the TCR*α* and TCR*β* regions in a Gaussian process method to determine which CDRs are important for epitope recognition. Similarly, ERGO utilizes LSTM and autoencoder models on multiple input features including the TCR*α* and TCR*β* sequences as well as the V and J genes for each TCR (11).

The major challenge for this computational problem is that the number of epitopes documented in these databases is far smaller in comparison to the actual number of epitopes arising in nature. In addition to this, interesting novel epitopes continuously rise, such as neoepitopes from cancer, and ones from new strains of pathogens. Epitope-specific models such as TCRGP and TCRex cannot *be used* for a novel or rare epitope because they can build a model for each epitope when a sufficient number of cognate TCRs are available. General models such as NetTCR, ERGO and our own ATM-TCR can make predictions for novel or rare epitopes but they all perform poorly (see Section 3.2-3.3). In order to address this issue, a computational model should be designed to learn cues that pinpoints the underlying biology of binding. Additionally, more efforts in embedding interpretability in model design should be given in order to provide further clues about novel epitope’s binding affinity.

In this paper, we present ATM-TCR, a novel computational solution to predict binding affinity between TCR and epitope. It uses a multi-head self-attention network (12) to obtain contextual representations of each sequence by considering how amino acid residues interact with each other within TCR and epitope sequences. Such learned representations may be more relevant in determining the physical binding logistics between the two molecules. One of the network outputs, called the *attention map*, provides interpretable information about how amino acid residues are correlated with each other. This information can be used to further validate the binding prediction score. We trained our model on a dataset of TCR*β* CDR3 and epitope sequence pairs where positive pairs were collected from the three databases and negative pairs were generated by random recombination of existing positive pairs. We show that the prediction performance of ATM-TCR outperforms the state-of-the-art binding affinity prediction models (improvements of 2% in AUC and 6.3% in recall). We also present a novel use of attention maps obtained from our model as a binding confidence score to further improve the model’s prediction on rare or out-of-sample epitopes using SARS-CoV-2 dataset (improvements of 41.11% in accuracy and 25% in recall).

## 2 Materials and Methods

### 2.1 Data and Preprocessing

Our dataset consists of binding TCR-epitope pairs collected from publicly available databases: VDJdb, McPAS-TCR, and IEDB. The collected data was processed into a unified format and then filtered down to pairs which contain human MHC class 1 epitopes and TCR*β* sequences. Additional quality control filters were applied to the remaining data. Pairs with linear epitope sequences were retained. Pairs containing wildcards such as * or X in sequences were also filtered out. Pairs reported in VDJdb have a confidence score that describes the verification processes used to ensure that pair binds. This confidence score ranges from 0-3 indicating low to high confidence. A confidence score of 0 indicates that a critical aspect of sequencing or validation for that pair is missing, so we removed pairs with a score of 0.

As summarized in **Figure 1B**, after the initial data processing and quality control, the resulting dataset consisted of 6,388 VDJdb pairs, 11,936 McPAS-TCR pairs, and 169,223 IEDB pairs. This was further reduced to a total 150,008 binding TCR-epitope pairs after removal of duplicates. We further filtered the dataset to keep only pairs where the epitope consisted of 17 or fewer amino acids as it has been suggested that linear epitopes only consist of up to 17 amino acids (13). This resulted in the *primary dataset* containing 128,142 unique TCR-epitope pairs, with 931 unique epitopes and 119,984 unique TCRs. We also created a *secondary dataset* of 150,008 binding pairs that included epitopes longer than 17 residues to test if those longer epitope sequences cause any significant performance changes. It contains 982 unique epitopes and 140,675 unique TCRs. Further details about the primary and secondary datasets are outlined in **Table 1**. Since TCR-epitope pairs known to not bind are not readily available, we generated negative pairs using random recombination. The primary and secondary datasets both consisted of a 1:1 ratio of positive and negative data (see Section 2.2 for more details).

**Table 1:** Data summary.

Name	Source	Unique Epitopes	Unique TCRs	Unique TCR-epitope Pairs	Overlapping Pairs with IEDB
Primary and Secondary	VDJdb^*^	187	3,915	4,047	1,208 (29.85%)
	McPAS-TCR	301	9,822	10,156	5,526 (54.41%)
	IEDB	1,189	136,492	145,678	145,678 (100%)
	Total (Primary)^**^	931	119,984	128,142	
	Total (Secondary)^***^	982	140,675	150,008	
Out-Of-Sample	IEDB (SARS-CoV-2)	2	332	332	

^*^VDJdb pairs with the score larger than 0 were used. ^**^Total number of epitopes, TCRs, and TCR-epitope pairs prior to pairs with epitope sequences greater than 17 amino acids in length being filtered out. ^***^Total number of unique epitopes, TCRs, and TCR-epitope pairs in union of the three sources without the length filter. The number of unique epitopes, TCRs, and TCR-epitope pairs in each source, and their overlap with IEDB.

In addition to the primary and secondary datasets used to build and evaluate our model, we additionally sourced an out-of-sample dataset from IEDB to evaluate a more generalized performance and to analyze benefits of using the self-attention mechanism. This dataset consisted of 332 TCR-epitope binding pairs from two unique SARS-CoV-2 epitopes not present in either the primary or secondary datasets. The first epitope, YLQPRTFLL, has 304 cognate TCRs and the other epitope, RLQSLQTYV, has 28 cognate TCRs.

### 2.2 Negative Sample Generation

We generated negative pairs *via* random recombination of the binding pairs with the same epitope distribution. For each positive pair, a negative pair is created by replacing the TCR with a randomly selected TCR from the same dataset. If the newly created pair is already present in the dataset, it is discarded and the process is repeated until a unique pair is generated. This creates a 1:1 ratio of binding and non-binding pairs. The exception to this rule is the out-of-sample (SARS-CoV-2) dataset which contained a limited number of epitopes. To generate non-binding pairs, TCRs from VDJdb are randomly selected and recombined with the out-of-sample epitopes.

### 2.3 Training and Testing Set Splitting

There is a tremendous number of novel TCRs and epitopes that have not been documented. Generalized prediction performance on novel TCRs and epitopes, however, cannot be properly measured by random splitting of training and testing sets. Random splitting usually generates training and testing sets which have common TCRs and epitopes, hence it can overestimate prediction performance. In order to precisely measure the prediction performance on unseen TCRs and epitopes, we designed two strategies to split the data into training and testing sets.

#### 2.3.1 TCR Split

The data is split such that any TCRs in the testing set are not found in the training set, meaning a TCR in the testing set has never been seen by the model. This strategy aims to evaluate prediction performance on out-of-sample TCRs. In order to split samples, a list of unique TCRs in the data is created and randomly divided into five distinct groups. Each TCR group is used to split samples into training and testing sets. For example, for the first fold, all TCR-epitope pairs whose TCRs belong to the first group are used as the testing set and all other pairs as the training set. All the other folds are created in a similar fashion.

#### 2.3.2 Epitope Split

The data is split such that any epitopes in the testing set are not found in the training set, meaning an epitope in the testing set has never been seen by the model. This strategy aims to evaluate prediction performance on out-of-sample epitopes. The epitope split is made similarly to the TCR split while using a list of unique epitopes instead of unique TCRs.

Since the number of cognate TCRs for each epitope varies and vise versa, the strategies do not always result in identically sized folds but produce the folds having similar sizes. Additionally, due to the amount of unique TCRs, the TCR splits tends to be similar to a random split of the data in practice.

### 2.4 Multi-Head Self-Attention Model for Binding Affinity Prediction

ATM-TCR, our binding affinity prediction model, consists of two encoders separately for TCR and epitope sequences and a linear decoder for determining the binding affinity between the two encoded sequences (**Figure 1D**). The most distinctive characteristic of our model is the multi-head self-attention mechanism (12) used by the two encoders to process TCR and epitope sequences. The mechanism was originally proposed in the natural language processing (NLP) field to learn contextual representations of words by accounting for their interactions within a sentence. ATM-TCR takes advantage of the mechanism to learn contextual representations of each amino acid residue within a sequence. The new representation of an amino acid residue is obtained by a weighted average over the other residues based on how strongly they interact with it. This helps the model learn the interactions among amino acids within TCR and epitope sequences by considering both the residue itself and positional information. Such learned representation is meaningful as residue interaction is the key to peptide-protein interaction.

Before the training, each TCR and epitope sequence is aligned using IMGT methodology (14) as shown in **Figure 1C**. The methodology uses two hyperparameters representing the maximum length allowed for TCR and epitope sequences, denoted as *p_T_* and *p_E_* respectively. If a sequence is shorter than the maximum, it adds paddings to the middle of the sequence until the maximum length is achieved. If a sequence is longer than the maximum, it removes amino acids from the middle of the sequence until the maximum length is achieved. These alignments become the inputs to ATM-TCR described in **Figure 1D**. Each amino acid in the aligned sequences is encoded as a one-hot vector. The one-hot encoded sequences are fed into an initial embedding layer to compute a sequence of embedding vectors with the size *p*. The sequence embeddings T ⋲ *R^p_T_^*^×^*^p^* and E ⋲* R^p_E_^*^×^*^p^* are then fed into corresponding encoders, which are denoted as *f_t_* and *f_e_* respectively. Each encoder uses multi-head self-attention mechanism that quantifies the strength of relationships between amino acids in a sequence and learns a contextual representation of the TCR and epitope. The new representation is given by an average of (linearly transformed) input embeddings weighted by the strength of the relationship. This process is illustrated in **Figure 1E**. In detail, a TCR sequence T is fed into three linear layers returning *key* (K) *query* (Q), and *value* (V) matrices as follows:


$$K=TW^{K},Q=TW^{Q},V=TW^{V}$$

where W*^K^*,W*^Q^*, and W^V^ are *p×p* matrices of which each element is a model parameter to be learned. The strength of relationship between *i*-th amino acid and the other residues, denoted as w*_i_* ⋲ *R^pT^*, is determined by the scaled dot-product of the *i*-th row of with all rows of as follows:


$$w_{i}=Softmax\left(\frac{q_{i}K^{T}}{\sqrt{p_{T}}}\right)$$

where q*_i_* is the *i*-th row of Q. W is defined by a matrix having w*_i_* for the *i*-th row vector and called as *attention map*. The contextual representation of the *i*-th amino acid is then defined as a linear sum of all amino acid vectors weighted by the attention map.


$$t_{i}^{*}=w_{i}V=w_{i1}v_{1}+\cdots +w_{ip_{T}}v_{p_{T}},T^{*}=WV$$

where w*_i_* is the *i*-th row of W. Each element of w*_i_* reflects an amino acid’s relative importance for determining the new representation of the *i*-th amino acid. Similarly, an epitope sequence is processed through the epitope encoder to obtain a new representation matrix. Such attention mechanism is called self-attention, and the new representation obtained by the mechanism is called a *self-attention feature*. Multi-head self-attention allows multiple self-attention heads to work in parallel and attend to sequence positions differently to spread out cognitive load of the attention mechanism. The multiple self-attention outputs are then concatenated, flattened, and fed into a single dense layer. Finally, the TCR (or epitope) encoder returns a sequence of representation vectors for the TCR (or epitope).

Then, the decoder *f_d_* determines the binding affinity between the TCR and epitope representation vectors. The encoded representation vectors, *f_t_* (T) and *f_t_* (E), are concatenated and fed into three linear layers each followed by a batch normalization layer, dropout layer, and activation (SiLU) function. The output of decoder is fed into a Sigmoid activation function to obtain a binding affinity score:


$$Score\left(T_{},E_{}\right)=\frac{1}{1+exp\left(-f_{d}\left(f_{t}\left(T\right),f_{e}\left(E\right)\right)\right)}$$

The binding score predicted by ATM-TCR is a continuous value between 0 and 1 where 0 represents non-binding and 1 represents binding for a given TCR and epitope pair.

### 2.5 Attention Map for Improving Out-of-Sample Prediction

In this section, we propose a novel approach to further validate and improve the out-of-sample prediction using attention maps of ATM-TCR. The attention map obtained from the TCR encoder enables the inferring of inter-relationships between amino acids in a TCR sequence. We assume that two TCR sequences have similar positional inter-relationships in their amino acid sequences if they bind to the same epitope. Those pair of TCRs, hence, are more likely to have similar attention maps. By extension, we assume the same for the pair of TCRs that bind to similar epitopes. On the other hand, if a pair of TCRs bind to two distant epitopes, they would have distinct attention maps. These assumptions were used to generate a new hypothesis. Previous predictions for epitopes with known cognate TCRs can be used to validate future predictions on similar out-of-sample epitopes by comparing attention maps of their TCR sequences.

**Figure 2:**
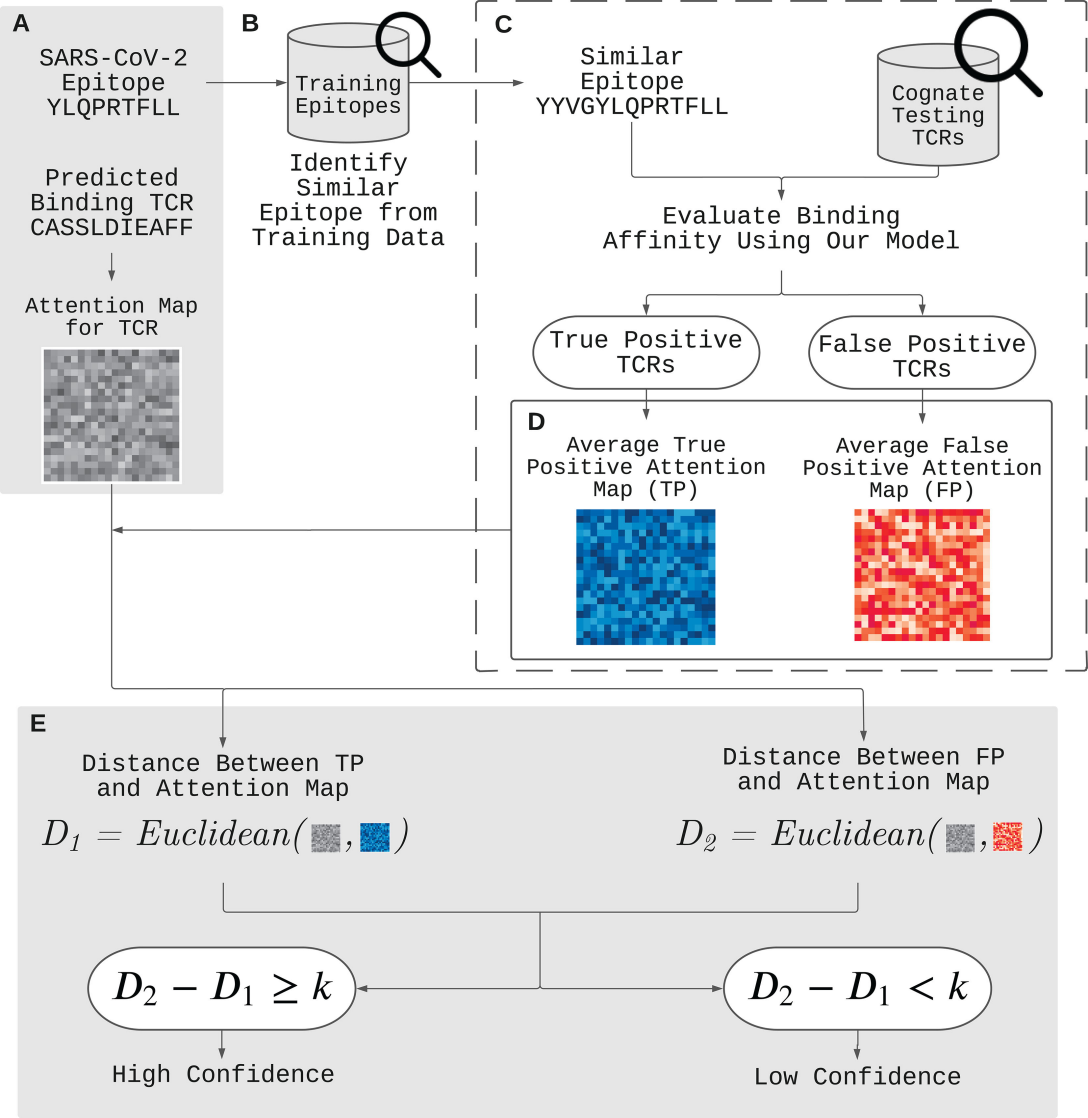
Improvement of out-of-sample prediction using attention maps. **(A)** We have a positively predicted (binding affinity score > 0.5) pair of a novel epitope (YLQPRTFLL) and TCR (CASSLDIEAFF) by ATM-TCR. In order to further validate the prediction, we utilize the attention map and determine its binding confidence level by comparing it with two reference attention maps for true and false positives. **(B)** We identify a reference epitope (YYVGYLQPRTFLL) from the dataset having the maximal longest common subsequence with the novel epitope. **(C)** We then evaluate the binding affinity between the reference epitope and each TCR in the data, and obtain the attention maps for the TCRs predicted as positive. **(D)** The TCRs are placed in two categories: one for true positive and another for false positive TCRs. The attention maps in each category are averaged together to create true positive and false positive reference attention maps. **(E)** If the Euclidean distance between the TCR’s (CASSLDIEAFF) attention map and the true positive representative map (***D*_1_**) is closer then that of the false positive representative map (***D*_2_**) it can be affirmed that there is high confidence that the positive prediction is correct. It follows that, if ***D*_2_ - *D*_1_ >= *k***, then we have high confidence in the positive prediction. On the other hand, if ***D*_2_ - *D*_1_< *k***, then we have low confidence in the positive prediction and it should be reconsidered as a false positive.

**Figure 2** demonstrates our approach to validate and improve the prediction using the attention map. Here is an example of such application. Given a positively predicted (binding affinity score > 0.5) pair of a novel epitope (YLQPRTFLL) and TCR (CASSLDIEAFF) by ATM-TCR, we want to further validate the prediction outcome. We first obtain the attention map of TCR (CASSLDIEAFF) from the TCR encoder (**Figure 2A**). The attention map is compared to that of true and false positive TCRs of an epitope similar to the novel epitope present in the dataset. This is denoted as *reference epitope*. We pick a reference epitope (YYVGYLQPRTFLL) from the dataset that has the maximal longest common subsequence with the novel epitope (**Figure 2B**). We evaluate the binding affinity of the reference epitope and TCRs present in the dataset using ATM-TCR (**Figure 2C**). The reference epitope’s paired TCRs are then sorted into one of four confusion matrix categories based on ATM-TCR’s binding affinity predictions and real values: true-positive (TP), false-positive (FP), true-negative (TN), and false-negative (FN) (**Figure 2D**). For each of these categories, a reference attention map is calculated by averaging the attention maps of TCRs in that particular category. Since the TCR (CASSLDIEAFF) is predicted to positively bind to the novel epitope, its attention map should be compared to the true-positive and false-positive reference maps. If the attention map of the TCR is similar to the true-positive reference map, then it can be affirmed that it is a binding TCR. If it shares more similarity with the false-positive reference map, it should be reconsidered as a non-binding TCR. As seen in **Figure 2E**, we use the Euclidean distance to calculate the similarity of the attention maps and reference maps. In order to systematically determine the similarity, we define *binding confidence score* as *D*_2_ - *D*_1_ where *D*_1_ is the Euclidean distance between a TCR attention map and the true-positive reference map, and *D*_2_ is that between a TCR attention map and the false-positive reference map. We determine there is high confidence that the positive prediction is correct if *D*_2_ - *D*_1_≥ *k* where *k* is a real value. On the other hand, if *D*_2_ - *D*_1_< *k*, then we have low confidence in the positive prediction and it should be reconsidered as false positive. Similarly, a TCR predicted as non-binding to the novel epitope should be compared to the true-negative and false-negative reference attention maps. It follows that, similarity with the true-negative matrix will affirm the prediction as non-binding, while similarity with the false-negative matrix indicates the TCR should be reconsidered as binding. In other words, if *D*_2_-*D*_1_≥ *k*, then the negatively predicted TCR should be reconsidered as false negative.

### 2.6 Hyperparameter Tuning

We optimized the model with the following hyperparameter search space (the bold indicates the choice for our final model): embedding matrix initialization – {Blosum45, Blosum50, Blosum62, **Random**}, padding– {Left, **Middle** Right}, dropout rate for the first dense layer – {**50%**, 60%}, dropout rate for the second dense layer – {**25%**, 30%, 50%}, the maximum length limit for TCR – {10, 15, 18, **20**, 22}, the maximum length limit of epitope – {10, 15, 18, 20, **22**}, the number of heads in the attention layer – {1, **5**}, the dimensions of the first dense layer – {256, 512, 1024, **2048**, 4096}, and the dimensions of the second dense layer – {256, 512, **1024**, 2048}. We used the Adam algorithm (15) with the learning rate of 0.001, the batch size of 32, the coefficients to compute running averages of the gradient (*β*_1_, *β*_2_) = (0.5, 0.999), and its square ϵ = 10^-8^. All models were trained for 200 epochs. We tuned the hyperparameters *via* exhaustive grid search and chose the hyperparameter values that yield the best AUC on the validation sets of 5-fold nested cross validation as shown in **Figure S1**. ATM-TCR is implemented using PyTorch, an open source deep learning platform (16).

### 2.7 Baseline Methods

We compared ATM-TCR against state-of-the-art binding affinity prediction models. Namely we trained three models: NetTCR (8), ERGO-AE, and ERGO-LSTM (11). All three of the models were trained on our collected datasets using the best performing hyperparameters reported in their corresponding literature. The same TCR and epitope splits were also utilized across all models to ensure consistency of training and testing sets between the models. The performance for each model is reported as an average of their AUC, recall, and precision across the 5 folds.

### 2.8 Statistical Tests

In this section, we enumerate and detail the statistical tests performed in our analysis. In Section 3.2-3.3 and 3.5, we performed the one-sided paired t-test to statistically compare the prediction performance (AUC, recall, and precision) between ATM-TCR and the other models. The performances were measured from the 5-fold testing sets, so the sample size for the test was 5. In the first test, a smaller p-value (< 0.05) means our model significantly performs better than the others. In the second test, a smaller p-value (< 0.05) means our model significantly performs worse than the others. If none of the tests yielded significant p-values, we concluded that no significant difference between two models was found. In Section 3.2, we performed the Pearson correlation test to measure the association between each epitope’s log-scaled frequency and the epitope-specific AUC. The epitope frequency was calculated from the primary dataset. The epitope-specific AUC was calculated as the average AUC of epitope in the TCR-split of the primary dataset. A smaller p-value (< 0.05) and greater correlation coefficient indicate epitope-specific AUC and frequency are significantly correlated. In Section 3.4, we performed the one-sided t-test to compare the binding confidence scores between the true and false positive (or negative) predictions on the out-of-sample dataset. A smaller p-value (< 0.05) indicates a significant difference in the binding confidence scores between the true and false positive predictions. In Section 4.5, we performed the two sample t-test to compare AUC of our models trained on the primary and secondary datasets to assess the effect of the epitope length filter that removed epitope sequences greater than 17 amino acids on the prediction performance of the models. A small p-value (< 0.05) indicates a significant difference in the prediction performances.

## 3 Results

### 3.1 Data

**Figure 3:**
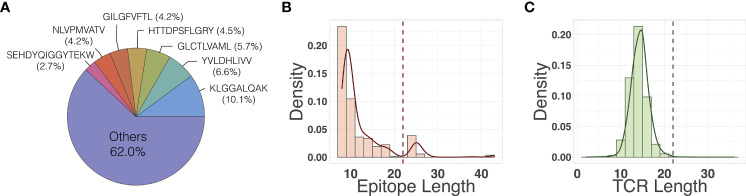
**(A)** Frequency of epitopes in the primary dataset. Distribution of **(B)** epitope length and **(C)** TCR length in the primary dataset. Dashed vertical lines indicate the maximum input sequence length hyperparameters chose by our model.

We compiled the *primary dataset* (128,142 unique binding pairs) and the *secondary dataset* (150,008 unique binding pairs) sourced from VDJdb, McPAS-TCR, and IEDB (see Section 2.1 for details). The primary dataset was further refined from secondary dataset by filtering out long epitope sequences (> 17 amino acids long). **Figure 3B** shows several outlier epitopes longer than 17, which were filtered out. All epitopes are at least 8 amino acids long. We did not filter the TCRs as no outliers were observed (**Figure 3C**). The *out-of-sample* dataset had 332 unique binding pairs, with two SARS-CoV-2 epitopes that are not present in the primary or secondary dataset. The primary dataset was used to train models and perform most of the analysis, unless otherwise specified. The secondary dataset was used to compare the performance of the models on data that included the longer sequence lengths to determine if the quality control process had any significant impact on the model performance, reported in Section 3.5. Finally, we used the out-of-sample dataset to evaluate the applicability of the model’s attention map for improving the binding affinity prediction, reported in Section 3.4. As shown in **Figure 3A**, a few epitopes were far more frequently observed than others in the primary dataset: 38% of the pairs in the data contain one of seven epitopes, each of which has more than 3,000 examples of binding TCRs within the dataset.

### 3.2 ATM-TCR More Accurately Predicts the Binding Affinity of Unseen TCRs Than the State-of-the-Art Models

**Figure 4:**
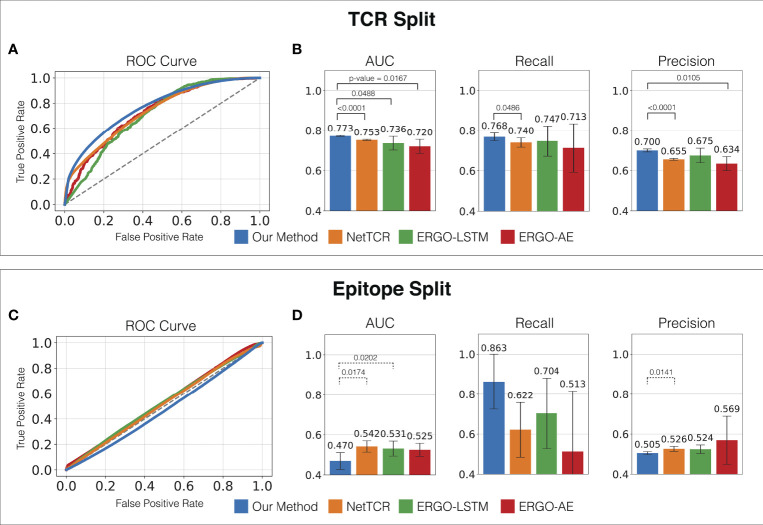
Prediction performance of ATM-TCR and all other methods measured on the primary dataset. **(A)** ROC curve, **(B)** AUC, recall, and precision of the TCR split. **(C)** ROC curve, **(D)** AUC, recall, and precision of the epitope split. The averages across the 5-fold testing sets were reported. For each fold Youden’s Index was utilized on the ROC curve to determine the optimal cut-off point to measure recall and precision. One-sided paired t-test was performed to test if each AUC, recall, and precision of ATM-TCR was significantly greater than the others, or the others were significantly greater than ATM-TCR. When ATM-TCR was significantly better, we reported the p-value and indicated the two methods with solid line above the bar plots. When the others were significantly better, we reported the p-values and indicated the two methods with dashed line above the bar plots. If none of the directions were significant, we did not indicate.

In order to evaluate the prediction performance for unseen TCRs, we trained and evaluated the models on the TCR split (TCRs in the testing set do not appear in the training set, see Section 2.3 for more details). As seen in **Figures 4A, B**, ATM-TCR achieves the highest average AUC, recall, and precision among all the models in binding affinity prediction for unseen TCRs. We statistically evaluated the performance differences using the one-sided paired t-test, and observed our method significantly outperforms all the other methods in AUC and precision, and NetTCR in recall with 0.05 significance level (i.e., 95% confidence level).

We observed ATM-TCR more accurately makes binding affinity predictions for highly frequent epitopes than for rare epitopes. In our dataset, several epitopes were observed in less than 10 pairs while others were observed in more than 10,000 pairs (**Figure S3**). The most frequently observed epitopes with > 10,000 pairs usually achieved higher AUCs—the average of the top six most frequent epitopes was 0.842 which is higher than the overall AUC of 0.773 (**Table S1**). We statistically assess the association between an epitope’s prediction performance and frequency using the Pearson correlation test. The test was performed on the (log-scaled) frequency and the epitope-specific AUC obtained by averaging the epitope’s AUC in the 5-fold TCR-split testing sets. The AUC and (log-scaled) frequency showed a significant linear relationship with a Pearson correlation coefficient of 0.852, and a p-value of < 2.2 ×10^-16^ as seen in **Figure S4**. A better prediction performance is expected for a more frequent epitope because the training set has richer information about them. The rare epitopes were poorly predicted because the training set does not have enough information about them. This is a common limitation of typical machine learning prediction models. Unlike other methods compared, ATM-TCR’s attention mechanism provides attention maps for TCRs and epitopes, allowing us to perform additional analysis to address such limitations. An application of the attention mechanism of ATM-TCR is demonstrated in Section 3.4.

**Figure 5:**
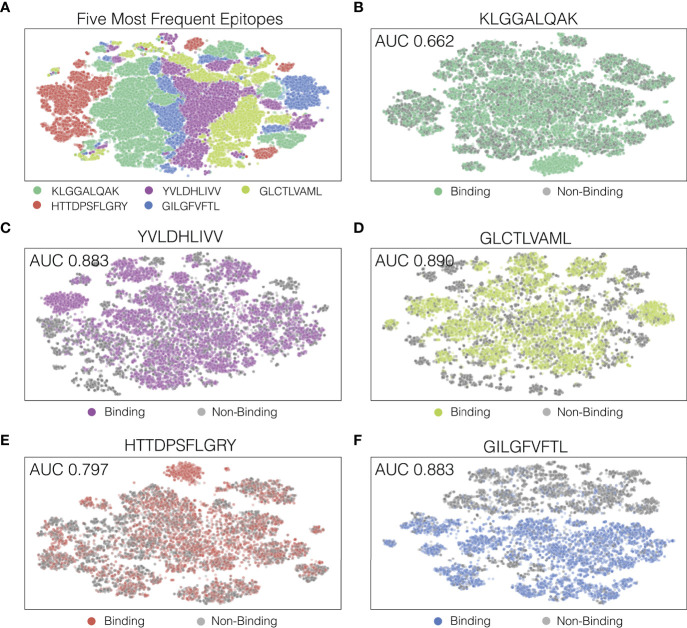
Visualization of ATM-TCR’s last hidden layer using t-SNE plots. Each dot indicates a TCR-epitope pair (either binding or non-binding). Panel **(A)** displays the t-SNE plot of the five most frequently appearing epitopes and the rest of the panels display t-SNE plots for **(B)** the most frequent epitope KLGGALQAK, **(C)** the second epitope YVLDHLIVV, **(D)** the third epitope GLCTLVAML, **(E)** the fourth epitope HTTDPSFLGRY, and **(F)** the fifth epitope GILGFVFTL.

In order to look at how our model separated decision boundaries, we performed t-SNE (17) on the output of the last hidden layers of the TCR-epitope pairs. **Figure 5A** shows a t-SNE plot for the five most frequently appearing epitopes, and **Figure 5B** through **F** each shows a t-SNE plot for the five epitopes individually with binding and non-binding pairs indicated as different colors. As seen in **Figure 5A**, the final hidden layer of ATM-TCR shows clear separation between pairs that have different epitopes. This is likely because epitopes sequences in the dataset were distinct from each other. However, t-SNE plot of individual epitope reveals several clusters and decision boundary was complex (**Figures 5B–F**). This aligns with our observation from hyperparameter tuning, that a more complex network achieves better performance. The t-SNE plots also show that the epitope-specific performances and decision boundaries vary in epitope. The decision boundary of GILGFVFTL is clear cut (AUC 0.937) while that of KLGGALQAK is not (AUC 0.662). Similar trends are observed from all the other models (**Table S2**) the performances vary in epitope and the models perform best on GILGFVFTL and worst on KLGGALQAK.

Although identical TCR-epitope pairs are not in both training and testing sets, there are TCRs binding to an identical epitope that share ranging sequence similarities. Highly similar TCRs binding to an identical epitope across training and testing sets may cause overestimation of prediction performance because models may be able to infer information more easily, when testing TCRs share higher sequence similarities to training TCRs. We assessed how ATM-TCR performs when removing TCRs that share sequence similarity from the TCR split testing sets in the primary data while gradually changing the minimum sequence similarity threshold value. The sequence similarity of each testing TCR was measured by (1) the minimum hamming distance between the testing TCR and all training TCRs (2), average of the top 10 lowest hamming distances, and (3) average of the top 5 lowest hamming distances. We gradually increased the threshold value *d* = 1, ⋯, 6, to remove all TCRs of hamming distances smaller than or equal to *d* from the test sets. The measured performances (AUC) for varying threshold values are shown in **Figure S5** The performance measure decreases along with the threshold and becomes stable at *d* = 4 and beyond. This indicates that the prediction performance of unseen TCRs differs by level of similarity with training TCRs.

### 3.3 All Models Including ATM-TCR Perform Poorly on Binding Affinity Prediction of Unseen Epitopes

In order to measure prediction performance for unseen epitopes, we trained and evaluated the models on the epitope split (epitopes in the testing set do not appear in the training set, see Section 2.3 for more details). All models including ATM-TCR failed to achieve reasonably good performance on the epitope split. As shown in **Figures 4C, D**, each model performs similarly to a random classifier with an AUC score around 0.5. Notably, all AUCs degraded on the epitope split compared to the TCR split. This may be because the training and testing data were more dissimilar to each other in the epitope split than in the TCR split. As shown in **Table 1**, the number of unique TCRs (119,984) is much larger than the number of unique epitopes (931) and together they make up 128,142 TCR-epitope pairs. This indicates that epitopes have many binding TCRs and most TCRs appear once (bind to single epitope) in the dataset.

### 3.4 ATM-TCR Can Improve the Poor Generalization Performance for Novel Epitope Using Attention Map

ATM-TCR, as well as the other models, demonstrated limited performance on the epitopes rarely observed or never been observed in the training sets. This inability to make predictions on rare or unseen epitopes was also mirrored by ATM-TCR’s results on the out-of-sample SARS-CoV-2 data, showing a recall of 50.00%. In this section, we demonstrate that the attention map obtained from the TCR encoder can help us to further validate predictions and hence improve the poor generalization performance. Our idea is based on the assumption that TCRs binding to similar epitopes would have similar inter-relationships between amino acids, and hence the model would have similar attention mechanism on those TCRs.

**Figure 6:**
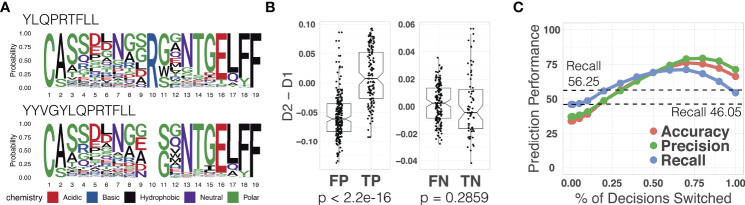
**(A)** TCR sequence logos binding to the novel epitope YLQPRTFLL and the reference epitope YYVGYLQPRTFLL. **(B)** The binding confidence score (***D*_2_ - *D*_1_**) of TCRs predicted as positive (left) and negative (right) in regard to the SARS-CoV-2 novel epitope YLQPRTFLL. The average scores of false positive and true positive TCRs showed a significant difference (p-value < **2.2 × 10^-16^**), while those of false negative and true negative showed no significant difference (p-value = **0.2859**), indicating that the binding confidence score can be used to further distinguish the false positive and true positive TCRs in out-of-sample prediction. **(C)** Prediction performance of the novel novel epitope YLQPRTFLL achieved by changing ATM-TCR’s decision using the confidence score. The x-axis represents the percentage of samples for which we changed the decisions and y-axis represents the prediction performance (accuracy, precision, and recall). When x is 70% (***k* = -0.01**) it achieved the best performance improvement in recall. This improved out-of-sample recall of 46.05% to 71.05%.

We used the out-of-sample epitope YLQPRTFLL as an example. Our TCR split model achieved an epitope-specific recall of 46.05% for 664 TCRs (332 binding TCRs collected from the out-of-sample dataset and 332 non-binding TCRs collected from the primary dataset). In order to improve the poor prediction, we followed the process illustrated in **Figure 2** (see Section 2.5 for details). We identified a reference epitope YYVGYLQPRTFLL from the primary dataset having the maximum longest common subsequences with the novel epitope YLQPRTFLL. We indeed observed that they were binding to similar TCR sequences as shown in **Figure 6A**. We generated reference attention maps for (1) true positive (2), false positive (3), true negative, and (4) false negative TCRs. In order to validate the prediction on the out-of-sample epitope, we calculated the binding confidence score *D*_2_ - *D*_1_ of 664 TCRs by comparing their attention maps to the reference maps.

The confidence score of the true positive TCRs of the novel epitope was significantly higher than the false positive TCRs (p-value < 2.2 × 10^-16^, paired t-test) as shown in **Figure 6B**. This means that the confidence score can be used to distinguish the true positives from the false positive. We observed that the negatively predicted TCRs showed slight score improvement in the false negatives than the true negatives, although it was not statistically significant. These results gave us a hint to use the confidence score to further improve the prediction performance. We switched the positive prediction to negative if the score is smaller than *k* (i.e., *D*_2_-*D*_1_< *k*). Note that in order to avoid all predictions from being changed from positive to negative, we also switched the negative prediction to positive if the score is equal to or larger than *k* (i.e., *D*_2_-*D*_1_ ≥ *k*). We measured the accuracy, precision, and recall values by gradually changing the threshold *k* that controls the number of switched decisions (*k* = 0.01, 0.05, 0.1, 0.2, 0.3, 0.4, 0.5, 0.6, 0.7, 0.8, 0.9, 1). In **Figure 6C**, we observed all three metrics gradually increased as we switched decisions using the attention map. The original recall value of 46.05% for our model increased to 71.05%. This value is even higher than the recall value (56.25%) we obtained solely based on the attention map.

### 3.5 ATM-TCR Performs the Best on the Secondary Dataset Including Epitopes Significantly Longer Than Others

Our dataset, which was sourced from the three databases, had several epitopes whose lengths are longer than the others as seen in **Figure 3B**. The primary dataset, that most of analysis was performed on, filtered out epitopes consisting of longer than 17 amino acids. In order to assess if the longer epitope sequences affect the binding affinity prediction process, we additionally trained all the models on the secondary dataset which contained all epitopes regardless of length, and statistically compared to those of the primary data. As shown in **Figure S2**, ATM-TCR also showed the best AUC and was significantly better than NetTCR and ERGO-LSTM in the TCR split in similar fashion to its performance on the primary dataset. In the epitope split, all models had no significant differences and failed to achieve reasonably good performance as they did on the primary dataset.

## 4 Discussion and Conclusion

ATM-TCR is a computational model to predict whether a TCR-epitope pair bind to each other. Predicting a TCR’s binding affinity to a target epitope is the fundamental step to select a confident set of potential therapeutic TCRs for T cell engineering (18, 19). Our study is in line with previously proposed computational models to predict binding affinities of TCRs to a target epitope, and eventually provide TCR candidates to expedite the development of adaptive treatments. The most distinctive component of our model is the multi-head self-attention mechanism. It allows us to selectively attend amino acids in a sequence based on how strongly they are correlated with each other. Consequently learning the biological contextual representations of TCRs and epitopes whose structure and function are determined by how amino acids are arranged and correlated with each other. We evaluated our computational model on both in-sample (TCR split and epitope split) and out-of-sample (SARS-CoV-2) data and compared it to state-of-the-art methods. We observed that ATM-TCR outperforms the state-of-the-art models for the TCR split by up to 2% in AUC and 6.3% in recall. We also showed that the learned attention map of our model can be used as a confidence measure which can improve the recall of our predictions on the out-of-sample dataset by up to 25%.

Many TCRs that bind to an identical epitope displayed high similarities, differing only by one or two amino acid residues. Therefore, in the TCR split, the binding TCRs of an identical epitope are likely to appear both in testing and training sets, allowing the models to easily infer information about the testing set from the training set. However, in the epitope split, the models struggled to make accurate predictions from the information given from the training epitope. Most epitopes in the entire dataset were distinct from each other with almost no sequence similarity, making it difficult for models to extrapolate the information learned from training data to epitopes in testing data. This trend is in line with our observation about the rare epitopes that were poorly predicted.

However, we observed the previously proposed models and our model suffer from poor prediction performance on rare or out-of-sample epitopes. To improve this performance, positive (or negative) predictions made by ATM-TCR were further validated by comparing their attention maps to the true and false positive (or negative) reference maps. The reference maps were obtained from a reference epitope to that of the out-of-sample epitopes. To determine the reference epitope, we identified the longest common subsequences (LCS) between each of the epitopes in the dataset and the out-of-sample epitopes, and used the epitope with the longest LCS as a reference epitope. We observed that our approach using the attention map can significantly improve out-of-sample performance. However, a sequence with an LCS longer than a few amino acids may not exist in a user dataset. This potentially limits the extent of this analysis until a greater amount of epitopes have been recorded or an alternative epitope similarity measure is utilized.

Three hyperparameters had the greatest effect on model performance (1): the dimension of the dense layer (2), number of heads in the attention layer, and (3) the maximum length limit of TCR and epitope. In particular, the combination of a large size of the dense layer and multiple attention heads greatly benefited the model’s performance, meaning ATM-TCR can be further improved if we use a more complex structure. Increasing the model capacity, however, can be detrimental to the generalization performance unless it is trained on a greater number of samples than currently collected. Additionally, ATM-TCR benefited from having length bounds on sequences. Reducing either the TCR or epitope sequence size below 15 amino acids, however, was very detrimental to model performance. We also observed that initializing the embedding with a BLOSUM matrix often had little to no effect compared to utilizing a random initialization. The performance boosts were often minimal, and no single BLOSUM matrix consistently outperformed other matrices or the random initialization method. The performance improvement by the binding confidence score was affected by a choice of *k*. We observed that the best improvement was at k ≈ 0, hence we recommend users to use the threshold 0 if no empirical experiment is available to determine the best value.

We also trained NetTCR-2.0 (20), the latest version of NetTCR. However, it performed worse than NetTCR: AUCs for the TCR and epitope split were 0.7283 and 0.5052, respectively (average of the 5 folds). It may be because NetTCR-2.0 was designed for TCR sequences having both the TCR*α* and TCR*β* chains, but our data includes only TCR*β* chain. The architecture for NetTCR-2.0 is extremely similar to NetTCR. The only difference is a slight dimension change of the layer to account for the additional information for TCR*α* chain. We focused on TCR*β* chain to secure enough training data and mitigate overfitting problems. Only 15% of our data has both TCR*α* and TCR*β* chain, which is not enough to train neural network models having a large number of parameters.

There are several components of ATM-TCR that can be improved in future. One potential area of improvement is that most existing models including ATM-TCR utilize a naive alignment method (IMGT method, see **Figure 1C**) that inserts paddings to the middle of each sequence until it reaches a fixed parameter length. Consequently, TCR and epitope sequences are always aligned the same way regardless of the other sequence it is aligned against. Changing this padding method to align relevant sections of the TCR sequence to its paired epitope sequence using biological domain knowledge would aid in training and predictions. Embedding positional information of amino acids, for example, using positional encoding (12) may alleviate any problems rising from paddings. We also saw improved AUC performance in the attention model when training and testing our model on sequences within the length limit of 17 or fewer amino acids. The TCR split showed significant AUC improvement (p-value < 0.0001) and the epitope split also showed AUC improvement (p-value < 0.170) but with no statistical significance. This performance boost was also noticeable in the other baseline models that we tested. This may indicate further curation of amino acid sequences is required in order to train the models on the longer sequences without performance degradation.

Furthermore, our method of obtaining the sequence representations may also be improved through use of sequence representation models such as DeepTCR (21). Since such models can be trained on a dataset of amino acid sequences with no binding information, we can use a large number of training samples and hence obtain more sophisticated representations of TCR and epitope sequences.

In summary, we proposed a computational model, ATM-TCR, to predict the binding affinity between a given pair of TCR and epitope sequences. Using attention as the primary structure of the model significantly increased the prediction performance compared to the state-of-the-art models. Furthermore, we demonstrated that the attention map of ATM-TCR can be used as an independent confidence measure for further correcting the out-of-sample predictions. Our model provides a more precise computational screening of a candidate pool of binding TCRs for a target epitope. Such screening ability can speed up the design process of identifying therapeutic TCRs in TCR gene therapy or other TCR engineering applications and is the first step toward towards making TCR therapy more accessible to the general public.

## SOFTWARE

The source code for the model is publicly available at https://github.com/Lee-CBG/ATM-TCR.

## Data Availability Statement

The datasets analyzed for this study are publicly available and can be found in VDJdb (https://vdjdb.cdr3.net), McPAS-TCR (http://friedmanlab.weizmann.ac.il/McPAS-TCR-TCR), and IEDB (https://www.iedb.org).

## Author Contributions

SB and HL contributed to conception and overall design of the study. MC, SB and HL designed the method and experiments. MC wrote the software and carried out the major experiments. MC, SB and PZ performed downstream analysis. MC, SB, and HL wrote the manuscript. All authors read and approved the final manuscript.

## Conflict of Interest

The authors declare that the research was conducted in the absence of any commercial or financial relationships that could be construed as a potential conflict of interest.

## Publisher’s Note

All claims expressed in this article are solely those of the authors and do not necessarily represent those of their affiliated organizations, or those of the publisher, the editors and the reviewers. Any product that may be evaluated in this article, or claim that may be made by its manufacturer, is not guaranteed or endorsed by the publisher.
