# Supplementary material for: ATM-TCR: TCR-Epitope Binding Affinity Prediction Using a Multi-Head Self-Attention Model
Source: Front Immunol. 2022 Jul 6;13:893247. doi: 10.3389/fimmu.2022.893247 (PMC9299376; doi:10.3389/fimmu.2022.893247)
Supplement: Supplementary file 1 [file DataSheet_1.pdf]

frontiers

Supplementary Material

Table S1. Epitope-specific prediction performance of ATM-TCR. Average AUC and standard deviation (SD) of the TCR split measured on the primary dataset are reported. Epitopes with frequency of $> 2,000$ are presented.

Epitope	AUC (± SD)	Frequency
KLGGALQAK	0.662 (± 0.002)	25880 (10.1%)
YVLDHLIVV	0.883 (± 0.005)	17022 (6.6%)
GLCTLVAML	0.890 (± 0.003)	14636 (5.7%)
HTTDPSFLGRY	0.797 (± 0.006)	11586 (4.5%)
GILGFVFTL	0.883 (± 0.004)	10802 (4.2%)
NLVPMVATV	0.937 (± 0.007)	10650 (4.2%)
SEHDYQIGGYTEKW	0.906 (± 0.011)	6848 (2.7%)
MGYINVFAFPFTIYSL	0.740 (± 0.013)	5864 (2.3%)
FVDGVPFVV	0.681 (± 0.012)	5420 (2.1%)
FLNGSCGSV	0.744 (± 0.022)	5136 (2.0%)
TPRVTGGGAM	0.699 (± 0.013)	5056 (2.0%)
KLSYGIATV	0.747 (± 0.018)	4920 (1.9%)
LLWNGPMAV	0.798 (± 0.018)	4716 (1.8%)
LPRRSGAAGA	0.669 (± 0.016)	4284 (1.7%)
LVVDFSQFSR	0.674 (± 0.014)	3750 (1.5%)
AELAKNVSLDNVL	0.701 (± 0.029)	3588 (1.4%)
APKEIIFLEGETL	0.693 (± 0.016)	3572 (1.4%)
LSPRWYFYYL	0.753 (± 0.011)	3502 (1.4%)
AVFDRKSDAK	0.605 (± 0.022)	3314 (1.3%)
KLPDDFTGCV	0.758 (± 0.025)	2638 (1.0%)
VLPFNDGVYFASTEK	0.687 (± 0.019)	2594 (1.0%)
IMLIIFWFSL	0.745 (± 0.024)	2562 (1.0%)

Table S2. Epitope-specific prediction performance of the five most frequent epitopes. Average AUC of the TCR split measured on the primary dataset are reported.

Epitope	ATM-TCR	netTCR	ERGO-LSTM	ERGO-AE
KLGGALQAK	0.662	0.604	0.647	**0.663**
YVLDHLIVV	**0.883**	0.778	0.878	0.844
GLCTLVAML	0.890	0.804	0.849	**0.891**
HTTDPSFLGRY	0.797	0.680	**0.836**	0.745
GILGFVFTL	**0.937**	0.812	0.854	0.912

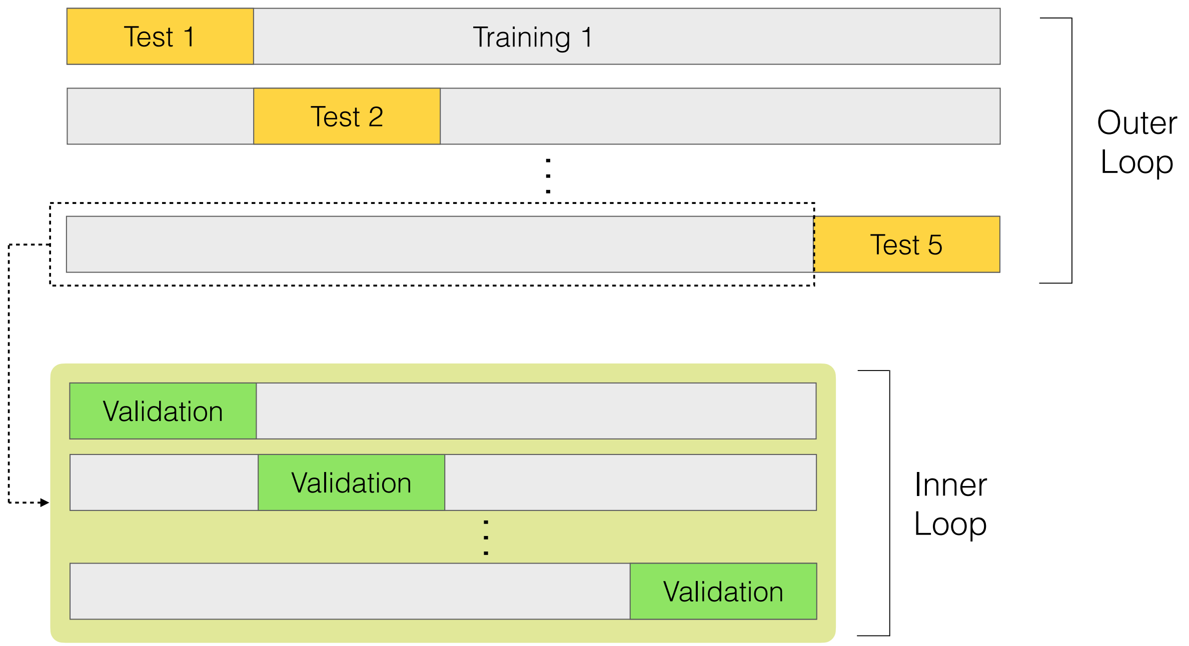

Figure S1: **Five-fold nested cross validation.** In the inner loop, models with different hyperparameter values are trained on the inner training sets, and tested on the validation sets. The hyperparameter that yields the best (average) validation AUC are chosen. For each the outer fold, the model with the best hyperparameter in the corresponding inner loop was trained on the outer training set, and evaluated on the test set. We report average AUC, recall, and precision values of the five test sets.

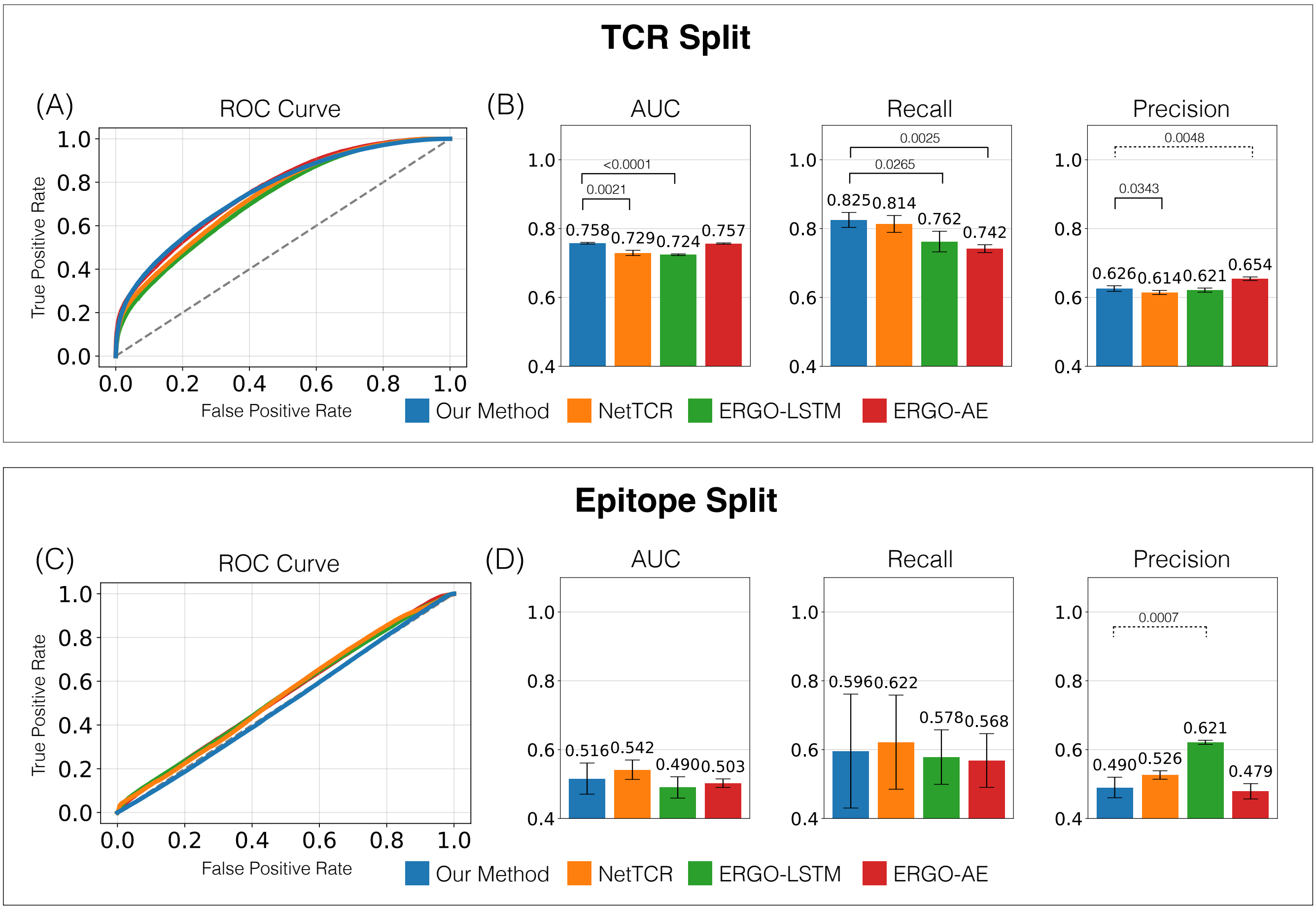

Figure S2: **Prediction performance of ATM-TCR and all other methods on the secondary dataset. (A)** ROC curve, **(B)** AUC, Recall, and Precision of the TCR split. **(C)** ROC curve, **(D)** AUC, Recall, and Precision of the epitope split. The averages across the 5-fold test sets are reported. For each model Youden's Index was utilized on the ROC curve to determine the optimal cut-off point to measure recall and precision. One-sided paired t-test was performed to test if each AUC, recall, and precision of ATM-TCR was significantly greater than the others, or the others were significantly greater than ATM-TCR. When ATM-TCR was significantly better, we reported the p-value and indicated the two methods with solid line above the bar plots. When the others were significantly better, we reported the p-values and indicated the two methods with dashed line above the bar plots. If none of the directions were significant, we did not indicate.

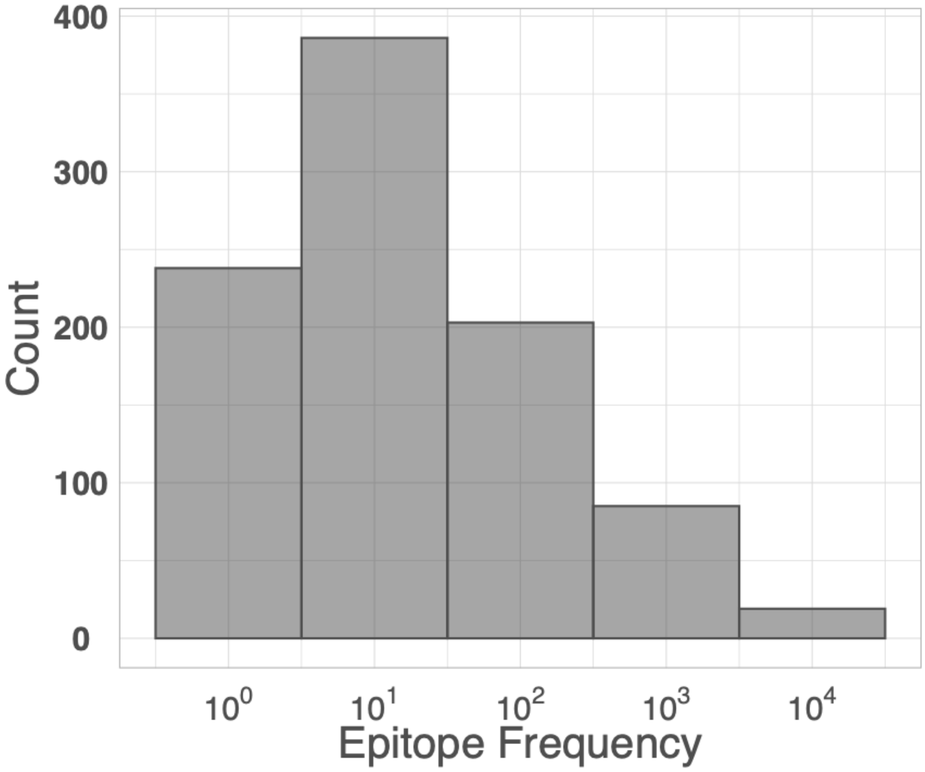

Figure S3: **Epitope frequency distribution of the primary dataset.**

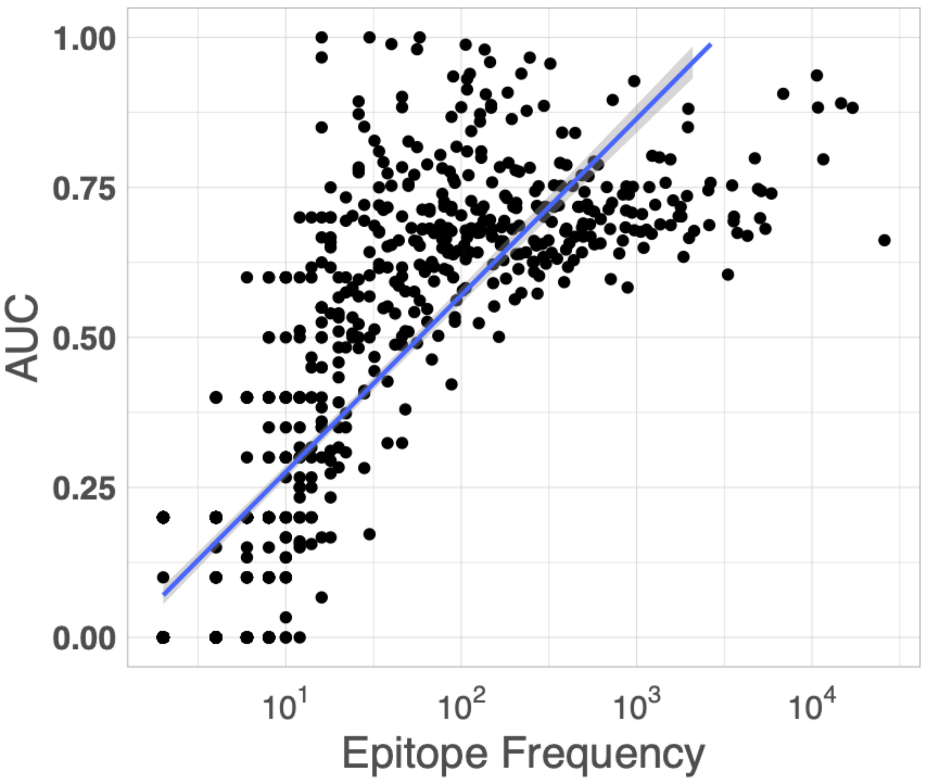

Figure S4: **Epitope-specific AUC versus its frequency.** The TCR split models trained on the primary dataset were used, and average AUC of the 5-fold test sets for each epitope is plotted with a simple linear regression line (blue) between the AUC and log-scaled frequency. Pearson's correlation coefficient between the AUC and the log-scaled frequency was 0.852. P-value of Pearson correlation test was $< 2.2 \times 10^{-16}$, meaning the correlation was significant (i.e., correlation is not equal to 0).

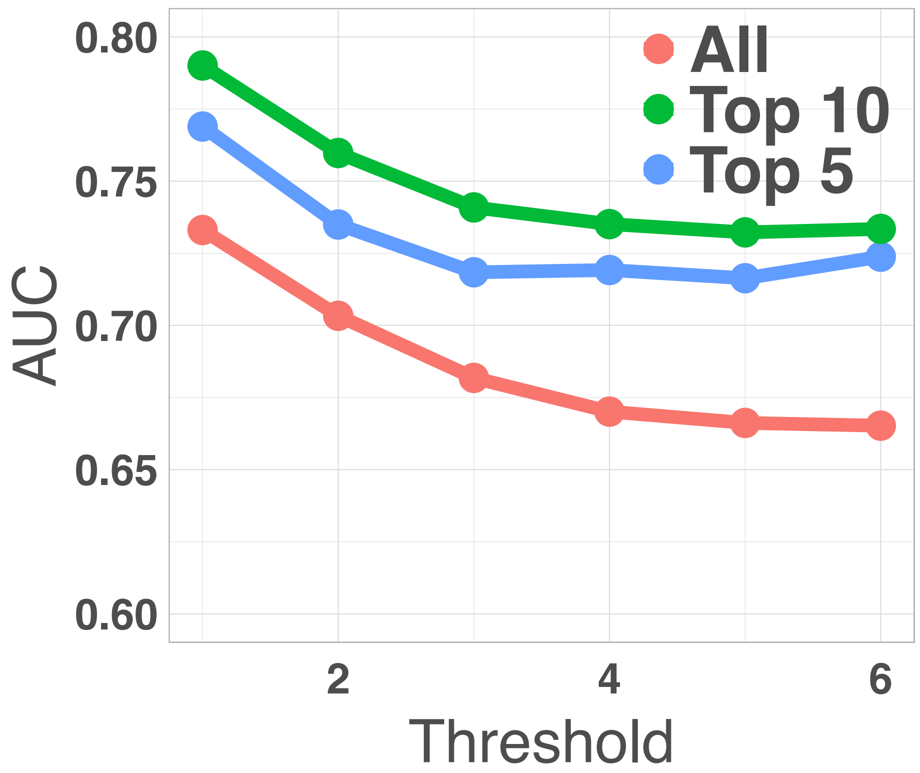

Figure S5: **AUCs along with the threshold** ($d = 1, \cdots, 6$) **for Hamming distance of a testing TCR to training TCRs.** The distance of a testing TCR is measured by (1) the minimum hamming distance between the testing TCR and **all** training TCRs, (2) average of the **top 10** lowest hamming distances, and (3) average of the **top 5** lowest hamming distances. A larger threshold indicates greater dissimilarity between training and testing TCRs of the TCR split.
